# Toward Safer Opioid Prescribing in HIV care (TOWER): a mixed-methods, cluster-randomized trial

**DOI:** 10.1186/s13722-022-00311-8

**Published:** 2022-05-16

**Authors:** Gabriela Cedillo, Mary Catherine George, Richa Deshpande, Emma K. T. Benn, Allison Navis, Alexandra Nmashie, Alina Siddiqui, Bridget R. Mueller, Yosuke Chikamoto, Linda Weiss, Maya Scherer, Alexandra Kamler, Judith A. Aberg, Barbara G. Vickrey, Angela Bryan, Brady Horn, Angela Starkweather, Jeffrey Fisher, Jessica Robinson-Papp

**Affiliations:** 1 Department of Neurology, Icahn School of Medicine at Mount Sinai, One Gustave L. Levy Place, Box 1052, New York, NY 10029 USA; 2 Department of Medicine, Division of Infectious Diseases, Icahn School of Medicine at Mount Sinai, New York, USA; 3 Department of Population Health Science and Policy, Icahn School of Medicine at Mount Sinai, New York, USA; 4 Department of Psychology and Neuroscience, University of Colorado Boulder, Boulder, USA; 5 Caring Accent (Consultancy), San Jose, CA USA; 6 Department of Economics and the Center On Alcoholism, Substance Use and Addictions, University of New Mexico, Albuquerque, USA; 7 Center for Evaluation and Applied Research, New York Academy of Medicine, New York, USA; 8 School of Nursing, University of Connecticut, Storrs, CT USA; 9 Institute for Collaboration On Health, Intervention, and Policy (InCHIP), University of Connecticut, Storrs, CT USA

**Keywords:** Chronic pain, HIV, Opioids, Opioid prescribing guidelines

## Abstract

**Background:**

The 2016 U.S. Centers for Disease Control Opioid Prescribing Guideline (CDC Guideline) is currently being revised amid concern that it may be harmful to people with chronic pain on long-term opioid therapy (CP-LTOT). However, a methodology to faithfully implement the CDC guideline, measure prescriber adherence, and systematically test its effect on patient and public health outcomes is lacking. We developed and tested a CDC Guideline implementation strategy (termed TOWER), focusing on an outpatient HIV-focused primary care setting.

**Methods:**

TOWER was developed in a stakeholder-engaged, multi-step iterative process within an Information, Motivation and Behavioral Skills (IMB) framework of behavior change. TOWER consists of: 1) a patient-facing opioid management app (OM-App); 2) a progress note template (OM-Note) to guide the office visit; and 3) a primary care provider (PCP) training. TOWER was evaluated in a 9-month, randomized-controlled trial of HIV-PCPs (N = 11) and their patients with HIV and CP-LTOT (N = 40). The primary outcome was CDC Guideline adherence based on electronic health record (EHR) documentation and measured by the validated Safer Opioid Prescribing Evaluation Tool (SOPET). Qualitative data including one-on-one PCP interviews were collected. We also piloted patient-reported outcome measures (PROMs) reflective of domains identified as important by stakeholders (pain intensity and function; mood; substance use; medication use and adherence; relationship with provider; stigma and discrimination).

**Results:**

PCPs randomized to TOWER were 48% more CDC Guideline adherent (p < 0.0001) with significant improvements in use of: non-pharmacologic treatments, functional treatment goals, opioid agreements, prescription drug monitoring programs (PDMPs), opioid benefit/harm assessment, and naloxone prescribing. Qualitative data demonstrated high levels of confidence in conducting these care processes among intervention providers, and that OM-Note supported these efforts while experience with OM-App was mixed. There were no intervention-associated safety concerns (defined as worsening of any of the PROMs).

**Conclusions:**

CDC-guideline adherence can be promoted and measured, and is not associated with worsening of outcomes for people with HIV receiving LTOT for CP. Future work would be needed to document scalability of these results and to determine whether CDC-guideline adherence results in a positive effect on public health.

*Trial registration* https://clinicaltrials.gov/ct2/show/NCT03669939. Registration date: 9/13/2018

## Background

Since 1999 over 840,000 Americans have died of drug overdoses [1]. For the first 15 years of this epidemic, prescription opioids were responsible for the majority of these deaths [2], and in response, significant attention was paid to reformation of opioid prescribing practices. The *U.S. Centers for Disease Control (CDC) Guideline for Prescribing Opioids for Chronic Pain* [3] (CDC guideline) was published in the *Journal of the American Medical Association* (JAMA) in 2016 and despite some initial controversy [4], was generally praised as a major acheivement, and a needed correction to overly liberal opioid prescribing practices [5, 6]. In the ensuing years, access to prescription opioids was reduced due to changes in the behavior of individual prescribers and pharmacies [7–9], as well as larger scale policies instituted by third-party payers and regulatory authorities [10, 11]. Although these changes were often based on the content of the CDC Guideline, there were important inconsistencies. For example, a recommendation to carefully assess risk and benefit before *increasing* opioid doses above 50 mg morphine equivalents (MME) and avoiding *increasing* above 90 MME, was implemented by some states as strict dosing limits, with some as low as 30 MME [10]. Extensive anecdotal and some quantitative data have since suggested that these changes have had a negative impact on people living with chronic pain (CP), particularly those on long term opioid therapy (LTOT) [12–14]. Moreover, in the years since the CDC Guideline was published presciption opioid mortality has been significantly outpaced by a sharp rise in deaths attributable to ilicitly manufactured fentanyl [2]. In 2021, a revision to the 2016 CDC Guideline was begun and the AMA released an open letter to the CDC, stating that “the 2016 Guideline is hurting patients” and that this is “a direct result of the arbitrary thresholds on dose and quantity.” [14] The revised guideline, which as of this writing is available in draft form for public comment, is substantively similar to the 2016 version but with the addition of specific language detailing how it should not be interpreted.

Given this context, a careful consideration of the purpose of opioid prescribing guidelines (i.e., what outcomes are desirable and reasonable to expect) and strategies for opioid guideline implementation and evaluation are needed. Without such efforts the effect of the CDC Guideline on individual patient outcomes and public health cannot be known, and attempts at reform will be inherently limited.

The present study was a demonstration project funded by the Agency for Healthcare Research and Quality (AHRQ) in 2017 with the goal of understanding how to implement and test the effect of the original 2016 CDC Guideline on patients with CP receiving LTOT focusing on people living with HIV. Although the CDC Guideline also addresses new opioid prescriptions/acute pain we did not include these patients because the care processes are substantively different, and because we anticipated that the approach to CP-LTOT represented the greater need. At the time of study design, the events described in the previous paragraphs were not yet known, however, we had anticipated that the CDC Guideline could be implemented in ways that might worsen outcomes by disrupting patient-provider relationships, particularly in vulnerable patient populations. This was one reason why we chose to focus on HIV-primary care, i.e. because of the high prevalence of medical, psychiatric and substance use co-morbidities, and risk factors for healthcare disparities (e.g., high prevalence of racial/ethnic minority and lower socioeconomic status). Moreover, CP in people living with HIV is common and associated with disability [15], reduced quality of life [16], risk behaviors such as heroin use [17], and suboptimal retention in care and virologic control [18].

We first engaged with patient and prescriber stakeholders to develop a CDC Guideline implementation strategy (termed TOWER) and then conducted a mixed-methods, cluster-randomized controlled trial to assess the feasibility of TOWER and to determine whether a change in provider CDC Guideline adherence could be detected. A secondary aim of the trial was to evaluate a broad range of patient-reported outcome measures (PROMs) for their ability to detect the effects of changing opioid prescribing practices on HIV patients with CP-LTOT.

## Methods

### Overview of the Study Design

This was a 9-month, mixed-methods, cluster-randomized, controlled trial of the TOWER intervention. HIV primary care providers (PCPs) were randomized to TOWER vs. usual care control and assessed for CDCG adherence over time. Enrolled patients were people living with HIV and chronic pain who were prescribed opioids by an enrolled provider, and were monitored over time for pain control, opioid misuse, and other outcomes. Protocol details have been described previously [19], and are summarized in the following subsections.

### Development of the TOWER intervention

The 12 recommendations contained within the CDC Guideline are summarized in Table 1. Items 4 and 6 refer to opioid initiation and acute pain and so were not included in our approach which focused on CP-LTOT. TOWER was developed from these recommendations in 5-steps which have been described previously in a manuscript focused on intervention development and the description of the protocol for the study described herein [19]. We used the Information, Motivation, and Behavioral Skills (IMB) model of behavior change as a framework, which posits that behavior change depends on information as to why change is needed, motivation for change, and the skills to perform the new behavior [20]. In Step 1, we qualitatively analyzed data from “think aloud” interviews with nine prescribers as they interacted with preliminary TOWER materials which sought to operationalize the CDC Guideline recommendations and create a workflow, these results have been reported separately [21]. Two major needs were identified: (1) decreasing the time burden of collecting the patient data needed to weigh opioid risk and benefit; and (2) assistance with opioid-specific communication strategies.

**Table 1:** Summary of CDC Guideline

1. Non-pharmacologic and non-opioid pharmacologic therapies are preferred
2. Establish and measure goals for pain and function
3. Discuss benefits and risks and clinician and patient responsibilities for managing opioid therapy
4. Use immediate-release opioids when starting
5. Carefully reassess benefit/risk when considering increasing dosage to ≥ 50 morphine milligram equivalents (MME)/day; avoid increasing dosage to ≥ 90 MME
6. When opioids are needed for acute pain, 3 days or less will often be sufficient; more than 7 days will rarely be needed
7. Follow-up and re-evaluate risk of harm within 1–4 weeks of a dose increase and at least every 3 months otherwise; reduce dose or taper and discontinue if harm outweighs benefit
8. Evaluate risk factors for opioid-related harms. Consider offering naloxone
9. Check Prescription Drug Monitoring Programs (PDMP)
10. Use urine drug testing at least annually
11. Avoid concurrent benzodiazepine and opioid prescribing
12. Arrange treatment for opioid use disorder if needed

In Step 2, we addressed the first provider need by developing an opioid management app (OM-App) and progress note template (OM-Note). OM-App collected data (directly from the patient) which are not usually in the electronic health record (EHR) and/or data that are likely to change over time using a short messaging system (SMS)-based mobile health technology that delivered a daily text message to the patient containing a link to a rotating survey of 2–3 questions each day. This format was chosen to maximize usability in diverse populations [22]. The OM-App questions (see Appendix A) were derived from the CDC Guideline, and responses were organized in a dashboard accessible to intervention providers via any web browser (sample image is available in the manuscript describing the protocol) [19]. Due to resource limitations, the OM-App data could not be incorporated directly into the EHR, nor was it possible to provide a “single sign on” whereby the provider could use the same username and password for the EHR and the OM-App database. OM-Note (see Appendix B) provided decision support for the opioid risk–benefit assessment and organized the data needed to perform this assessment.

In Step 3, we engaged people living with HIV using the method of Public Deliberation to elicit their recommendations for opioid care processes and to specifically seek input on the communication challenges identified by providers in Step 1, this has been reported separately [23]. In Step 4 we synthesized these recommendations, developed a provider training, and sought and incorporated feedback from an additional 12 providers on our materials (OM-App, OM-Note and the training). The final training was a single, ~ 90 min, one-on-one session, given by the study principal investigator (PI). The session covered the content of the CDC Guideline, addressed the IMB barriers to CDC guideline adherence which had been identified in prior development steps, and oriented the provider to OM-App and OM-Note.

### Outcome measures

A validated method to quantify adherence to an opioid prescribing guideline in its entirety (rather than individual components such as MME or the use of urine drug testing (UDT)) did not previously exist and so we developed, validated and published the Safer Opioid Prescribing Evaluation Tool (SOPET) as our primary outcome measure [24]. The SOPET is used to score clinical documentation for CDC Guideline adherence, and produces a score from 0 to 15, where 15 indicates perfect adherence. The SOPET includes all items in the CDC’s published quality improvement measures for opioid prescribing [25] and outcomes used in prior studies (e.g. MME, UDT). The SOPET was scored twice for each patient-participant. Importantly, since the OM-Note was developed to facilitate CDC Guideline adherence, and the SOPET was developed to measure adherence to these same items, there is an inherent similarity.

The baseline SOPET score was taken from the patient’s last clinic visit with the PCP prior to enrollment. For the follow-up score, all PCP-patient visits in the 9 months following the patient’s enrollment were considered, and the visit with the most complete opioid-relevant documentation was chosen. We did this because often there were visits focused exclusively on medical issues in which opioids were not addressed.

With regard to patient-centered measures, our developmental work with patients and providers [19, 21, 23] identified six domains that might be impacted by changes in opioid prescribing practices: (1) pain intensity and function; (2) mood; (3) substance use; (4) medication use and adherence; (5) relationship with provider; (6) stigma and discrimination. The validated instruments we chose to reflect these domains are listed in Table 2. We also employed a novel composite dichotomous outcome at the end-of-study visit with a “successful” outcome defined as the satisfaction of all of the following criteria: no current opioid use disorder (OUD); no opioid overdoses during the study period; stable or improved pain control and function, defined as no clinically significant worsening (< 30% compared to baseline) in the Brief Pain Inventory (BPI); and (given our focus on the HIV care setting) an undetectable HIV-1 viral load indicating well-controlled HIV.

**Table 2:** Patient-reported Outcome Measures (PROMs) administered at baseline, 3-month, 6-month and 9-month follow-up

Construct	Instrument
Pain intensity and function	• Brief Pain Inventory (BPI) [43]
Mood	• Hospital Anxiety and Depression Scale (HADS) [44]
Substance use	• World Mental Health Composite International Diagnostic Interview (CIDI) substance use disorders module [45] • Current Opioid Misuse Measure (COMM) [46] • Self-Reported Misuse, Abuse, and Diversion (SR-MAD) [47]
Medication use and adherence	• Quantitative Analgesic Questionnaire (QAQ) [48] • AIDS Clinical Trials Group (ACTG) antiretroviral adherence questionnaire [49]
Relationship with provider	• Trust in Provider Scale (TIPS) [50] • Clinician & Group Consumer Assessment of Healthcare Providers and Systems (CG-CAHPS) survey (selected questions) [51]
Stigma and discrimination	• HIV Stigma Scale (HSS) [52] • Internalized Stigma of Chronic Pain (ISCP) [53] • Brief Perceived Ethnic Discrimination Questionnaire-Community Version (BPEDQ-CV) [54]

### Setting and recruitment

**Fig. 1:**
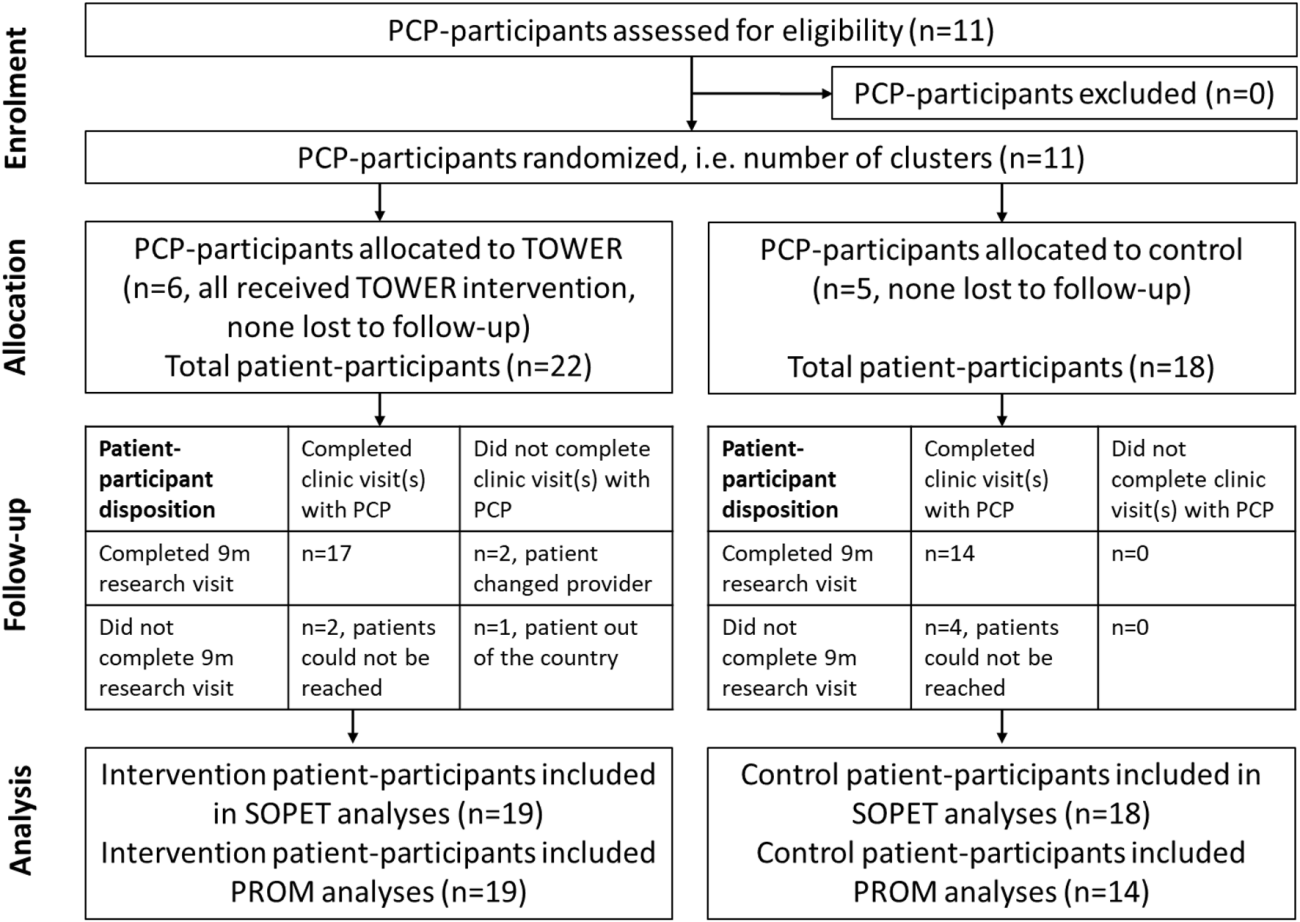
CONSORT diagram for cluster-randomized clinical trial

All procedures were performed according to a protocol approved by the Mount Sinai Institutional Review Board; and the trial was registered with ClinicalTrials.gov (NCT03669939). All participants provided written informed consent. The Mount Sinai Health System in New York City contains a network of five primary care clinics that care for approximately 10,000 people with HIV. All providers (physicians and nurse practitioners) who served as primary care providers (PCPs) in the clinic system and prescribed LTOT to at least five people with HIV for CP were eligible. Using the EHR, we generated lists of all PCPs and their patients who were prescribed LTOT. We then invited PCPs based on diversity (e.g., practice location within the system, gender, duration of practice) and patient volume, and contacted them individually by e-mail. Patient-participants were adults with HIV who were currently prescribed antiretroviral therapy and had at least 4 clinic visits over the past year (to reflect a generally stable engaged patient population), had opioids prescribed by an enrolled PCP for CP (≥ 3 months, not due to cancer or other terminal condition), spoke English with the PCP, and had access to a device capable of receiving messages from OM-App. For each PCP, we contacted sequential patients on their list and enrolled the first 5 eligible patients who agreed to participate. Figure 1 demonstrates participant flow through the study. The study was conducted from October 2018 to September 2020.

### Participant procedures

PCPs were enrolled by the study PI and randomized 1:1 to TOWER versus control (parallel groups) consecutively. PCPs were allocated to their group by selecting a sealed envelope, which enclosed a letter stating “control” or “intervention.” The letters were generated, and sealed and shuffled by study staff prior to any PCP enrollment. Control PCPs received no training, no decision support materials, and no access to OM-App data. Patient-participants had four visits with the research team (baseline, 3, 6, and 9 months) at which patient-reported outcome measures (PROMs) were administered (Table 2). All patient-participants were trained on the OM-App at baseline and educated about PCP access to their responses (yes for intervention patients, no for controls). Patient-participants received $50 for each research visit, and an additional $25/month if they completed ≥ 80% of the OM-App questions.

### Qualitative data collection

We performed two rounds of one-on-one, semi-structured interviews with PCPs. Baseline interviews were conducted by the PI to discover any additional IMB needs for CDC Guideline implementation. Exit interviews (with intervention providers only) were conducted by a health psychologist (co-author MCG) with the purpose of seeking feedback on TOWER. Specifically we sought to ascertain PCP confidence in carrying out individual CDC Guideline recommendations, and ways in which the OM-Note and OM-App did or did not support these efforts. We focused on the following areas: using non-pharmacologic treatments; discussing responsibilities for managing prescription opioids; ordering and interpreting UDT; ordering naloxone; accessing the prescription drug monitoring program (PDMP); making referrals for OUD treatment; and weighing the risks and benefits of prescription opioids. The interview guides were developed collaboratively by the study team (co-authors GC, MCG, LW, MS, AK, AB, JDF and JRP) based on instruments used in prior IMB studies (by co-author JDF) [26, 27]. Baseline interviews were documented in hand written notes. Exit interviews were audiotaped, professionally transcribed, and analyzed thematically as previously described [28]. In addition, one clinic visit for each patient-participant with their PCP was audiotaped, transcribed and qualitatively analyzed; due to the lengthiness of these qualitative results they have been described in detail separately, but are summarized herein where appropriate to provide context. [28].

### Statistical analyses

The original enrolment goal was 10 PCPs and 50 patient participants. This was not based on a power calculation because TOWER was a new intervention with a new outcome measure (SOPET). Rather this was intended as a feasibility study, i.e. a small-scale test of the methods and procedures needed to successfully demonstrate efficacy of the TOWER intervention (or other prescription opioid management strategies) in future adequately-powered randomized controlled trials. When fewer than 50 patient-participants were recruited from the first 10 PCPs, a plan was made to recruit additional PCPs, however this plan was terminated after one additional PCP due to the onset of the first large wave of COVID-19 in New York City in early 2020.

Data were summarized as mean ± standard deviation or median (interquartile range) for continuous variables and frequency (percentage) for categorical variables. Bivariate assessments of differences in demographic factors between the intervention and control groups at baseline were conducted using t-tests or Wilcoxon Rank Sum tests for continuous variables, and chi-squared or Fisher’s Exact Tests for categorical variables.

We compared the change in SOPET trajectories from baseline to follow-up between intervention- and control-patients using a mixed effects model with a random intercept. Generalized estimating equations with a logit link function (in an effort to account for repeated measures) were applied to the individual SOPET items, while adjusting p-values for multiple testing using the Benjamini and Hochberg method (Table 4) [29]. In exploratory analyses, we used mixed effects models with a random intercept to examine whether the trajectory differed between the intervention groups over time for the patient-centered measures (Table 5). Statistical significance was assessed at α = 0.05. All data analyses were conducted using SAS 9.4 and R Studio 1.3.1093. All audiotaped qualitative data were professionally transcribed and subjected to thematic analysis as previously described [30]. Participants with missing data were excluded from the relevant analyses. Specifically, there were three patient-participants who did not have a clinic visit with the PCP prior to the COVID-19 shutdown and so who could not be given a follow-up SOPET score (see also Table 4). There were also missing data for other outcome measures (see Table 5).

## Results

### Participant characteristics

All PCPs (ten physicians and one nurse practitioner) we approached agreed to participate, and the recruited sample (N = 11) was relatively diverse (6 women and 5 men; 5 White, 2 Black, and 4 Asian) with practice duration ranging from within 5 years of training to > 20 years in practice. Five PCPs were randomized to control, and six to TOWER. Patient-participant characteristics (N = 40) are summarized in Table 3; the control and intervention groups were similar with respect to age, sex, race and educational attainment, but differed in employment status with more patient-participants in the intervention group reporting that they were unemployed or unable to work. Overall patient-participants were adherent to antiretroviral therapy with 80% of participants reporting adherence of at least 95% and only two participants reporting adherence of less than 80%. Similarly most had well-controlled HIV, with only three participants with viral loads above 100 copies/ml at baseline. There were no significant differences between groups in antiretroviral adherence or virologic control. A total of 136 patients were contacted to enroll these 40. Reasons for not enrolling included: not being able to reach the patient (e.g. phone disconnected, or messages not returned); excessive burden of medical or other life issues; general disinterest in research; wanting to avoid extra travel; and technology-related issues (e.g. lack of smartphone or discomfort with text messages).

**Table 3:** Patient-participant characteristics^a^

	Overall (N = 40)	Control (n = 18)	Intervention (n = 22)	p-value
Age in years^b^	61 (7.3)	63 (9.1)	60 (5.5)	0.3
Sex				0.5
Women	22 (55%)	9 (50%)	13 (59%)	
Men	18 (45%)	9 (50%)	9 (41%)	
Race/ethnicity				0.2
Black/African-American	30 (75%)	16 (89%)	14 (64%)	
Hispanic/Latinx	8 (18%)	2 (11%)	6 (27%)	
Non-Hispanic White	2 (5%)	0 (0%)	2 (9%)	
Educational Attainment				1.0
Some high school or ≤ 8th grade	9 (23%)	4 (22%)	5 (23%)	
Completed high school or equivalent	12 (30%)	5 (28%)	7 (32%)	
Some college	10 (25%)	5 (28%)	5 (23%)	
Associates/Bachelor's/PG degree	9 (23%)	4 (22%)	5 (23%)	
Employment Status				0.03
Unable to work/Unemployed	26 (65%)	8 (44%) 7	18 (82%)	
Retired	9 (23%)	(39%)	2 (9%)	
Employed (full- or part-time) or Homemaker	5 (13%)	3 (17%)	2 (9%)	

^a^Values are n (percentage) unless otherwise indicated

^b^Value is mean (standard deviation)

### Primary outcome

**Fig. 2:**
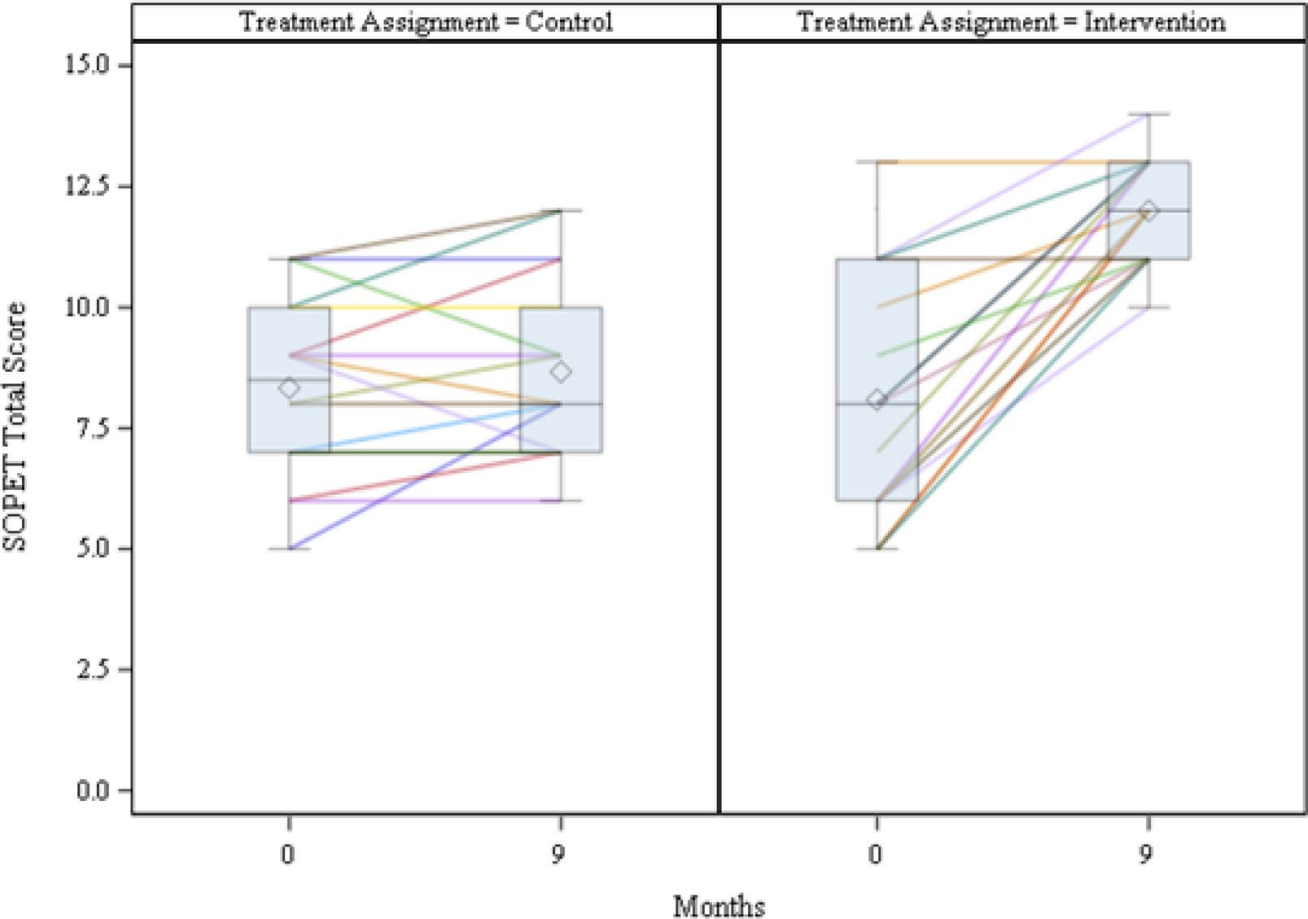
CDC opioid prescribing guideline adherence. Each line represents an individual patient-participant

The intervention group exhibited a significant increase in CDC Guideline adherence, as measured by SOPET scores, as compared to the control group (3.8, p =  < 0.0001, Fig. 2). Intervention-PCPs showed an average of 48% improvement in their SOPET scores; the mean SOPET scores for the intervention group (n = 19 patient-participant records) were 8.1 (SD = 2.5) at baseline and 12.0 (SD = 1.1) at follow-up, whereas controls (n = 18 patient-participant records) were 8.3 (SD = 1.8) at baseline and 8.7 (SD = 1.8) at follow-up.

Since the SOPET is based on EHR documentation, which may not accurately reflect the content of the encounter, we also performed qualitative analysis of audiotaped clinic visits between patients and PCPs. This has been reported separately but confirmed greater alignment with the CDC Guideline in the spoken content of the intervention PCPs’ visits with their enrolled patient-participants [30]. Adherence to individual aspects of the CDC Guideline is summarized in Table 4. Improvements were noted in: use of non-pharmacologic treatments, establishing a functional treatment goal and opioid agreement, assessment of opioid benefit/harm, use of the PDMP, and appropriate naloxone prescribing.

**Table 4:** Provider-participant adherence to individual CDC guideline elements, assessed by review of patient-participant electronic health records

	Baseline		Follow-up		p-value	Adjusted p-value
	Control (n = 18)	Intervention (n = 22)	Control (n = 18)	Intervention (n = 19)		
1. Non-pharmacologic treatment	8 (44%)	12 (54%)	7 (39%)	17 (90%)	0.01	0.018
2. Non-opioid pharmacologic treatment	10 (56%)	18 (82%)	12 (67%)	17 (90%)	0.7	0.14
3. Treatment goal	2 (11%)	2 (9%)	1 (6%)	14 (74%)	0.009	0.018
4. Discussion of patient opioid responsibilities or written opioid contract	4 (22%)	3 (14%)	4 (22%)	9 (47%)	0.01	0.018
5. Median MME (IQR)	24.75 (9.375, 101.25)	60 (45, 175)	24.75 (9.375, 101.25)	75 (40, 160)	0.2	0.26
6. Had visits with patient at least every 3 months	13 (72%)	16 (73%)	10 (56%)	14 (74%)	0.3	0.32
7. Assessed opioid benefit	7 (39%)	7 (32%)	9 (32%)	19 (68%)	< 0.0001	0.0003
8. Assessed opioid harm or risk of harm	16 (89%)	20 (91%)	17 (94%)	19 (100%)	< 0.0001	0.0003
9. Reviewed PDMP data	9 (50%)	11 (50%)	12 (67%)	19 (100%)	< 0.0001	0.0003
10. Performed urine drug testing	8 (44%)	14 (64%)	9 (50%)	15 (79%)	0.3	0.32
11. Patient not co-prescribed benzodiazepines	18 (100%)	19 (86%)	18 (100%)	16 (84%)	0.5	0.5
12. Follow-up planned within 1–4 weeks, if dose increased	18 (100%)	22 (100%)	18 (100%)	22 (100%)	N/A	N/A
13. Documented whether or not a high risk situation was suspected, and if so documented a plan to manage it	1 (5%)	5 (23%)	3 (17%)	18 (95%)	< 0.0001	0.0003
14. Low risk patient, or if high risk provided naloxone	9 (50%)	5 (23%)	9 (53%)	12 (63%)	0.03	0.048

### PCP-level qualitative data

Baseline provider interviews were conducted to assess whether there were additional IMB needs for CDC Guideline adherence. Minor changes to OM-Note were made in response to these data, otherwise the themes largely overlapped those from the earlier development work [31]. Exit interviews were conducted with five out of six providers who had been randomized to intervention (the sixth provider was not interviewed because she did not use the TOWER materials prior to shut-down of non-urgent outpatient care in New York City due to COVID-19 in March 2020). Exit interviews focused on the following areas: using non-pharmacologic treatments; discussing responsibilities for managing prescription opioids; ordering and interpreting UDT; ordering naloxone; accessing the PDMP; making referrals for OUD treatment; weighing the risks and benefits of prescription opioids; utilization of OM-App and OM-Note.

All PCPs reported regularly recommending non-pharmacologic treatment to their patients although barriers were reported including patient willingness and access: “*I mention things like weight loss, and then some rehabs, some PT, that kind of thing… I think in many cases they’re very dismissive of it”* and *“I think the frustration is where to go and how to get it paid for.”*

All PCPs also reported addressing patient responsibilities for managing prescription opioids, and four of five specifically mentioned counseling patients to keep opioids away from others, for example: *“Oh, yes, I discuss it with them, too, making sure that the medicine—do they live with a child, do they live with a teenager, they should make sure that it should always be in a safe location.”* One PCP noted that this was a change for her which she attributed to study participation: “*It’s probably not a conversation I’ve had on a regular basis, except because it is in the study template. …About how they are—the question about whether they live by themselves, whether other people have access to their medications, how they secure it.”*

All PCPs reported being confident in their ability to order and interpret UDT. Four out of five were comfortable ordering naloxone with two attributing this knowledge to study participation, for example: *“It was very easy… I think when I met with her, part of her whole training was she brought it and showed me the* [naloxone] *kit, and I’d never seen it before.”* All PCPs were comfortable using the PDMP, and had been so prior to study participation. All PCPs also had a plan for referring patients for OUD treatment if needed: *“I think it depends… Do they need inpatient? Is it outpatient? How can we troubleshoot it? That’s usually my first step”* and “*I would refer them out. We have a very robust program in our clinic that deals with this and I would walk them over to somebody who’s able to have this conversation in a more meaningful way with them than I can.”*

PCP confidence in weighing opioid risk and benefit was more mixed. Four of the five expressed some level of comfort but this was partial in some cases: *“I’d say I feel comfortable, but what I find harder about the risk/benefit with opiates is that the benefits are really patient measured and reported.”* The remaining PCP expressed ambivalence over whether managing opioids should be her role: *“I just feel like it came to me without much expertise and there’s a lot of—I guess—emotional things around whether I should be dealing with this or not, but I am.”*

Specifically with regard to the TOWER materials all PCPs reported finding the OM-Note useful: *“Very helpful, yes, because—…Well, it actually makes everything complete for that particular visit… No, no challenges at all, it was easy”* and *“That, I actually found pretty useful because I think it gave me bullet points. If I was gonna forget… I wouldn’t have given her Narcan script.”* Two providers commented on the length of OM-note, but also felt that it was likely unavoidable: *“I mean, it was slow. It was a trudge. But it wasn’t—I don’t know if it could be anything other than”* and *“So, we ended up discussing six other kind of medical issues so the note was just really sorta clunky with that template thrown in there. But otherwise, I mean, it’s pretty comprehensive.”* With regard to OM-App three out of five PCPs reported that the information was helpful: *“Looking at other ADLs and that was, to me, more meaningful—than just them rating their pain.”* However four out of five PCPs found lack of integration of the dashboard into the EHR burdensome: *“…too much time already, running late to go through all of this stuff and the text and calculate the morphine equivalence and give them a Narcan script if I had it. Then to also pull in the dashboard.”*

### Patient-level data

The overall patient response rate to OM-App was 70% (i.e. 70% of all daily questionnaires sent to all participants were answered). There was no evidence to suggest that some questions were answered more often than others, that participants tended to avoid any particular questions, or that adherence changed over time. There was no evidence of intervention-associated change (Table 5) in any of the patient-centered measures we used. Intervention-patients achieved the dichotomous outcome (stable or improved pain and function, no OUD or overdose, and undetectable viral load) more commonly than controls (47% vs. 33%, OR = 1.80) but this was not statistically significant (p = 0.6).

**Table 5:** Patient-reported outcome measure (PROMs) scores^a^

Outcome measure	Baseline		Follow-up	
	Control (n = 18)	Intervention (n = 22)	Control (n = 14)	Intervention (n = 19)
Current Opioid Misuse Measure	4.9 (4.5)	6.1 (6.3)	2.9 (3.1)	6.0 (4.8)
Brief Pain Inventory, Pain intensity	5.8 (2.1)	6.0 (1.6)	6.4 (1.7)	6.2 (1.9)
Brief Pain Inventory, Pain interference	4.3 (2.7)	4.0 (2.3)	3.3 (4.9)	4.1 (3.0)
Hospital Anxiety and Depression Score	10.2 (8.0)	11.9 (6.7)	8.8 (6.5)	10.9 (6.9)
Trust in Provider Score	82.3 (15.7)	80.0 (12.9)	78.8 (14.9)	77.0 (12.5)
Brief Perceived Ethnic Discrimination Questionnaire (Community Version)	0.7 (0.8)	0.5 (0.5)	0.7 (0.9)	0.6 (0.6)
HIV-associated Stigma Score	33.8 (11.0)	34.1 (8.2)	32.8 (7.1)	35.3 (6.9)
Internalized Stigma of Chronic Pain	64.6 (18.2)	64.2 (14.2)	62.4 (13.7)	(14.1)

^a^Values are mean (standard deviation). There are no statistically significant differences

## Discussion

Opioid prescribing guidelines, such as the CDC Guideline, were developed with the goal that provider adherence would lead to better patient and/or public health outcomes. However, establishing a strong evidence base to support these causal pathways is complex. First, a method to reliably change provider behavior toward greater guideline adherence must be developed including a means of measuring that adherence. Then it must be established that greater adherence mediates an effect on patient and/or public health outcomes, including determination of which outcomes are most important. We undertook the present study to test the first steps in this pathway, namely determining whether TOWER could lead to measurable effects on provider CDC guideline adherence in the HIV primary care setting. We also sought to establish a framework for testing the latter steps of the pathway in future work.

We found that providers randomized to TOWER were more CDC Guideline-adherent than those randomized to control. This was apparent in the primary outcome measure, the SOPET, which measured overall adherence based on EHR documentation and was corroborated by qualitative analyses. This is important because the great majority of past studies relied exclusively on unidimensional EHR-based outcomes (e.g. UDT, opioid contracts, MME) [32–41], which have the advantage of being relatively straightforward and objective but also have a limited capacity to capture the content of delivered care. We also found no change, either favorable or unfavorable, in the patient-centered outcomes we employed to reflect the concepts of pain intensity and function, mood, substance use, medication adherence, relationship with provider, stigma and discrimination. This suggests that the CDC Guideline can be implemented in such a way that it does not appear to cause harm to patients with CP on LTOT. These findings are similar to larger recent study, focused specifically on the HIV care setting, which found that the TEACH intervention (consisting of a nurse care manager with an interactive electronic registry, opioid education, academic detailing, and access to addiction specialists) met the primary outcome of increasing the odds of ≥ 2 UDTs during the study period, but did not have an effect on patient outcomes. [42].

Avoidance of harm is important, however, to be worthwhile, opioid prescribing guidelines should also produce benefit, and so it is necessary to define what outcomes are desirable and reasonable to expect. The potential beneficial outcomes of opioid prescribing guidelines as they pertain to CP patients already on LTOT can be broadly categorized into: 1) improving patient outcomes, and 2) minimizing harm to the community. With regard to improving patient outcomes, it is plausible that the CDC Guideline might improve pain and function by increasing provider attention to optimizing non-opioid and non-pharmacologic therapies, and encouraging realistic functional goal setting. It might also increase detection and treatment of OUD, and reduce fatal opioid overdoses by increasing naloxone prescriptions. We did not find evidence of an effect of CDC Guideline adherence on these outcomes in our study, nor did the recent TEACH study which enrolled 187 patients [42]. However, such an effect, if present, is likely to be small (given the modest efficacy of most treatments for chronic pain, and low incidence of new OUD in stable CP-LTOT patients) and therefore require a much larger sample size to detect. For example, assuming a similar cluster-randomized design, approximately 500 patient-participants would be needed to detect an effect size of 0.35 on pain outcomes with 80% power.

Minimizing harm to the community would entail reducing access to prescription opioids for non-medical users. There are two ways of accomplishing this: reducing the supply overall, and reducing the likelihood that opioids prescribed to patients are used by others. Reducing the supply overall appears to have been the dominant approach thus far, and the one which has been implicated as harmful to pain patients. The latter approach requires patient opioid stewardship behaviors such as refraining from giving opioids to others and keeping opioids secured. Since this is a patient behavior any effect of a guideline on it would necessarily be indirect (i.e., mediated by provider behavior). In Step 3 of this project during our Public Deliberation sessions with patient stakeholders we specifically discussed opioid stewardship behaviors, and patients recommended that providers educate patients about the dangers associated with opioid misuse and the importance of safe storage practices using empathic and open communication styles. Our qualitative data suggest that intervention-PCPs were doing this, however, our study was not designed to ascertain whether this would result in the desired patient behavior.

The limitations of this study are important in informing the design of future studies and clinical opioid guideline implementation efforts. The study had a relatively small sample size and was conducted in a single health system with patients who spoke English with their PCP. We obtained informed consent from every patient-participant (rather than seeking approval for a waiver of informed consent). This was necessary to obtain detailed PROMs but significantly curtailed enrollment (only 40 out of 136 eligible patients participated) and may have biased our sample, for example, by excluding participants wary of having their LTOT impacted by study procedures. The PCP training was conducted live and one-on-one by the study PI which would not be possible in a scaled up setting. Some opioid prescribing information is inherently local, e.g. resources for non-pharmacologic treatments, and could not be part of a disseminated intervention. We provided monetary incentives for study participation and OM-App adherence, to improve the likelihood of study success, however, doing so is unlikely to be sustainable in clinical practice. OM-App data were also likely underutilized by intervention-PCPs because of lack of EHR-integration, although we did not have the capability of directly tracking provider utilization patterns of OM-App. However EHR-integration is resource intense and likely impractical in many settings.

Perhaps the most important and instructive limitation pertains to the method of guideline adherence measurement. Prior studies have mostly used outcomes such as MME or UDT which have the distinct benefit of being simple and relatively objective. However, owing to this very simplicity, such outcomes cannot be used to convincingly support or refute a causal pathway between guideline adherence and patient outcomes. The primary outcome measure used in this study was the SOPET, which does reflect the CDC guideline more holistically. However, the improvements seen in SOPET score were likely directly related to the fact that the OM-Note prompted providers to document CDC guideline adherent behaviors. The SOPET alone cannot be used to establish that the provider actually performed the documented behaviors, although our qualitative audiotaped data (reported separately) did. Moreover, this approach did not allow us to adequately assess for decay of intervention effect over time because once the OM-Note was incorporated into the EHR, it tended to be copied forward in subsequent notes, leaving it unclear whether the topic matter was actually discussed at subsequent visits which were not audiotaped. Unfortunately, this weakness is not readily fixable because alternative strategies (e.g., direct observation of visits, solicitation of post-visit patient-participant feedback) were also problematic in that direct observation likely influences provider behavior and post-visit patient-participant feedback is subjective and was so challenging to obtain consistently that we ultimately abandoned it.

Despite these limitations, this demonstration project provides key and timely insights into the potential for real world use of the CDC Guideline in complex patient populations such as people living with HIV and how success might be judged. Specifically, our findings suggest that even in a complex patient population at risk for stigmatization and health disparities, the CDC Guideline does not appear to cause harm if faithfully implemented. This supports the approach that has been taken in the CDC Guideline revision which does not substantively alter the content but does provide additional specific instruction on how the content should and should not be implemented. Based on our findings and our experience during the study we have since adopted a simplified approach to roll out to our larger clinic community. The main tenets of this approach are: (1) avoidance of opioids whenever possible for CP in patients who are not currently on them; (2) use of the OM-Note to organize opioid management (see Appendix B); and (3) a suggested approach to weighing risk vs. benefit using the principles of harm reduction including: offering a trial of opioid taper to willing patients; avoidance of opioid taper in unwilling otherwise stable patients; and collaboratively-derived action plans for behaviorally unstable patients.

## Conclusion

In conclusion, this study demonstrates that, in the HIV care setting, prescriber adherence to the CDC Opioid Prescribing Guideline can be increased safely using the TOWER intervention which is comprised of a patient-facing opioid management app, a progress note template, and a prescriber training. Future pragmatic work will be needed to understand the impact of such an approach on a larger scale and in diverse care settings.

## Data Availability

Data from this study is available upon reasonable request.

## Appendix A. Opioid management app (OM-App) questions

Assessing benefit:

1. What number best describes your pain on average in the past week? 0 = no pain, 10 = pain as bad as you can imagine
2. What number best describes how pain has interfered with your enjoyment of life in the past week? 0 = does not interfere, 10 = completely interferes
3. What number best describes how pain has interfered with your general activity in the past week? 0 = does not interfere, 10 = completely interferes
4. Over the past week how often did you achieve your pain management goal? Never, rarely, sometimes, often, very often

Assessing harm and risk of harm (all reference the past week and are answered yes or no):

5. Have you experienced constipation?
6. Have you experienced nausea, vomiting, and/or lack of appetite?
7. Have you had trouble thinking clearly or memory problems?
8. Has anyone told you that you stop breathing during sleep?
9. Have you used alcohol?
10. Have you used drugs?
11. Have you found yourself craving or having difficulty controlling the use of your opioid pain medication?
12. Have you taken any opioid pain medications other than those prescribed by your primary care provider?
13. Have you needed medical attention because you took too much of your opioid pain medication?
14. Did anyone else take any of your opioid pain medications?

Assessing pain treatment utilization:

15. Did you do any of the following activities to manage your pain in the past week? Physical therapy, exercise, yoga, acupuncture, massage, meditation, something else, nothing
16. Please select your opioid pain medication from the list: buprenorphine, codeine, fentanyl patch, hydrocodone, hydromorphone, methadone, morphine, oxycodone, oxymorphone, propoxyphene, tapentadol, tramadol
17. How many pills (or patches) of this medication did you use in the last 24 h?

## Appendix B. Opioid management progress note (OM-Note) template

### Opioid management plan

The pain treatment goal is:

### Current pain treatment plan

- Current non-pharmacologic treatments:
- Current non-opioid pain medications are:
- Current opioid medications are:
- This corresponds to a maximum daily morphine mg equivalent of:
  **Table:**
  Assessment of opioid risk factors (should be established once and then updated as needed)			
  	Present	Absent	Notes
  Respiratory conditions			
  Renal insufficiency			
  Hepatic insufficiency			
  Pregnancy			
  Age 65 or older			
  Depression			
  Anxiety			
  Benzodiazepine use			
  Personal history of substance use disorder			
  Family history of substance use disorder			
  **Table:**
  Weighing benefit/risk/harm:	Yes	No	Action
  Opioid use disorder suspected (3 C’s: Loss of Control, Compulsive use, Continued use despite harm)?			If yes to one of these, prescribe naloxone, make addiction referral, taper opioids
  Recent overdose			
  Visible sedation or intoxication			
  Benefit of opioids?			If no, consider consensual taper
  Other opioid side effects?			If yes, consider more frequent monitoring

### Monitoring/risk mitigation:

- Urine drug testing (recommended at least once a year):
- Date checked: Results:
- PDMP check (recommended with every prescription):
- Date checked: Reference #: Results:
- Review of patient responsibilities (recommended at least annually): take opioids only as prescribed, do not increase the dose; do not get opioids from any other doctors; do not use alcohol or drugs; always keep opioids in a secure place, and never share them with others; contact me if you are concerned that opioid use disorder might be developing; do not miss appointments. Dates reviewed:
- Naloxone prescription: Y/N If yes, date prescribed.
- Patient stable (opioid dose stable, no concerns)? Y/N.
- If yes, follow-up ≤ 3 months.
- If no, follow-up 1–4 weeks.

## Abbreviations

AHRQ: Agency for Healthcare Research and Quality
AMA: American Medical Association
BPI: Brief Pain Inventory
CDC: Center for Disease Control
CP: Chronic pain
CP-LTOT: Chronic pain treated with long term opioid therapy/treatment
EHR: Electronic health record
IMB: Information, Motivation and Behavioral Skills
JAMA: Journal of the American Medical Association
LTOT: Long term opioid therapy/treatment
MME: Milligram morphine equivalents
OM-App: Opioid management app
OM-Note: Opioid management note
OUD: Opioid use disorder
PCP: Primary care provider
PDMP: Prescription drug monitoring program
PROM: Patient reported outcome measure
SMS: Short messaging system
SOPET: Safer opioid prescribing evaluation tool
UDT: Urine drug testing

## References

[1] Wide-ranging online data for epidemiologic research (WONDER). Centers for Disease Control and Prevention, CDC, National Center for Health Statistics. Updated 2020. Accessed Oct 2021, https://www.cdc.gov/drugoverdose/deaths/index.html

[2] Overdose Death Rates. Centers for Disease Control and Prevention, CDC, National Center for Health Statistics. Updated January 29, 2021. Accessed Oct 2021, https://www.drugabuse.gov/drug-topics/trends-statistics/overdose-death-rates

[3] Dowell D, Haegerich TM, Chou R (2016). CDC guideline for prescribing opioids for chronic pain-United States, 2016. JAMA.

[4] Olsen Y (2016). The CDC guideline on opioid prescribing: rising to the challenge. JAMA.

[5] Wen LS, Lloyd MC (2016). Prescribing opioids for chronic pain. JAMA.

[6] McAuliffe WE (2016). Prescribing opioids for chronic pain. JAMA.

[7] Goldstick JE, Guy GP, Losby JL, Baldwin G, Myers M, Bohnert ASB (2021). Changes in initial opioid prescribing practices after the 2016 release of the CDC guideline for prescribing opioids for chronic pain. JAMA Netw Open.

[8] Fenton JJ, Agnoli AL, Xing G (2019). Trends and rapidity of dose tapering among patients prescribed long-term opioid therapy, 2008–2017. JAMA Netw Open.

[9] Pedersen CA, Schneider PJ, Ganio MC, Scheckelhoff DJ (2020). ASHP national survey of pharmacy practice in hospital settings: prescribing and transcribing-2019. Am J Health Syst Pharm.

[10] Davis CS, Lieberman AJ (2021). Laws limiting prescribing and dispensing of opioids in the United States, 1989–2019. Addiction.

[11] Barnett ML, Olenski AR, Thygeson NM (2018). A health plan's formulary led to reduced use of extended-release opioids but did not lower overall opioid use. Health Aff (Millwood).

[12] Agnoli A, Xing G, Tancredi DJ, Magnan E, Jerant A, Fenton JJ (2021). Association of dose tapering with overdose or mental health crisis among patients prescribed long-term opioids. JAMA.

[13] Weeks WB (2016). Hailey. JAMA.

[14] AMA backs update to CDC opioid prescribing guidelines. American Medical Association. Updated July 22, 2021. Accessed Oct 2021, https://www.ama-assn.org/press-center/press-releases/ama-backs-update-cdc-opioid-prescribing-guidelines

[15] Merlin JS, Westfall AO, Chamot E (2013). Pain is independently associated with impaired physical function in HIV-infected patients. Pain Med.

[16] Sabin CA, Harding R, Bagkeris E (2018). Pain in people living with HIV and its association with healthcare resource use, well being and functional status. AIDS.

[17] Knowlton AR, Nguyen TQ, Robinson AC, Harrell PT, Mitchell MM (2015). Pain symptoms associated with opioid use among vulnerable persons with HIV: an exploratory study with implications for palliative care and opioid abuse prevention. J Palliat Care.

[18] Merlin JS, Long D, Becker WC (2018). Brief report: the association of chronic pain and long-term opioid therapy with HIV treatment outcomes. J Acquir Immune Defic Syndr.

[19] Robinson-Papp J, Aberg J, Benn EKT (2019). Decreasing risk among HIV patients on opioid therapy for chronic pain: development of the TOWER intervention for HIV care providers. Contemp Clin Trials Commun.

[20] Fisher JD, Fisher WA, Misovich SJ, Kimble DL, Malloy TE (1996). Changing AIDS risk behavior: effects of an intervention emphasizing AIDS risk reduction information, motivation, and behavioral skills in a college student population. Health Psychol.

[21] Navis A, George MC, Scherer M, Weiss L, Chikamoto Y, Robinson-Papp J (2019). What physicians need to implement safer opioid prescribing: a qualitative study. J Opioid Manag.

[22] Anderson-Lewis C, Darville G, Mercado RE, Howell S, Di Maggio S (2018). mHealth technology use and implications in historically underserved and minority populations in the United States: systematic literature review. JMIR Mhealth Uhealth.

[23] Scherer M, Weiss L, Kamler A (2020). Patient recommendations for opioid prescribing in the context of HIV care: findings from a set of public deliberations. AIDS Care.

[24] Navis A, George M-C, Nmashie A, Hoang E, Cedillo G, Robinson-Papp J (2020). Validation of the safer opioid prescribing evaluation tool (SOPET) for assessing adherence to the centers for disease control opioid prescribing guidelines. Pain Med.

[25] Shoemaker-Hunt S, Sargent W, Swan H (2020). Developing clinical quality improvement measures aligned with the CDC guideline for prescribing opioids for chronic pain: an important strategy to support safer prescribing in primary care. Am J Med Qual.

[26] Fisher JD, Fisher WA, Cornman DH, Amico RK, Bryan A, Friedland GH (2006). Clinician-delivered intervention during routine clinical care reduces unprotected sexual behavior among HIV-infected patients. J Acquir Immune Defic Syndr.

[27] Fisher JD, Cornman DH, Shuper PA (2014). HIV prevention counseling intervention delivered during routine clinical care reduces HIV risk behavior in HIV-infected South Africans receiving antiretroviral therapy: the Izindlela Zokuphila/Options for Health randomized trial. J Acquir Immune Defic Syndr.

[28] Scherer M, Kamler A, Weiss L (2021). Toward safer opioid prescribing: effects of the TOWER intervention on HIV care providers. AIDS Care.

[29] Benjamini Y, Hochberg Y (1995). Controlling the false discovery rate: a practical and powerful approach to multiple testing. J Roy Stat Soc: Ser B (Methodol).

[30] Scherer M, Kamler A, Weiss L (2021). Toward safer opioid prescribing: effects of the TOWER intervention on HIV care providers. AIDS Care.

[31] Navis A, George MC, Scherer M, Weiss L, Chikamoto Y, Robinson-Papp J (2018). What physicians need to implement safer opioid prescribing: a qualitative study. J Opioid Manag.

[32] Anderson D, Zlateva I, Khatri K, Ciaburri N (2015). Using health information technology to improve adherence to opioid prescribing guidelines in primary care. Clin J Pain.

[33] Liebschutz JM, Xuan Z, Shanahan CW (2017). Improving adherence to long-term opioid therapy guidelines to reduce opioid misuse in primary care: a cluster-randomized clinical trial. JAMA Intern Med.

[34] Quanbeck A, Brown RT, Zgierska AE (2018). A randomized matched-pairs study of feasibility, acceptability, and effectiveness of systems consultation: a novel implementation strategy for adopting clinical guidelines for Opioid prescribing in primary care. Implement Sci.

[35] Witt TJ, Deyo-Svendsen ME, Mason ER (2018). A model for improving adherence to prescribing guidelines for chronic opioid therapy in rural primary care. Mayo Clin Proc Innov Qual Outcomes.

[36] Huang KTL, Blazey-Martin D, Chandler D, Wurcel A, Gillis J, Tishler J (2019). A multicomponent intervention to improve adherence to opioid prescribing and monitoring guidelines in primary care. J Opioid Manag.

[37] Arizmendez NP, Kotovicz F, Kram JJF, Baumgardner DJ (2019). Multimodal local opioid prescribing intervention outcomes in chronic noncancer pain management. J Am Board Fam Med.

[38] Gupta A, Lindstrom S, Shevatekar G (2020). Reducing opioid overprescribing by educating, monitoring and collaborating with clinicians: a quality improvement study. Cureus..

[39] Marszalek D, Martinson A, Smith A (2020). Examining the effect of a whole health primary care pain education and opioid monitoring program on implementation of VA/DoD-recommended guidelines for long-term opioid therapy in a primary care chronic pain population. Pain Med.

[40] Stack M, Larouche V, Zhang Y, Warden D, Stack C, Klugiene EA (2020). Effects of implementing a comprehensive opioid reduction protocol on overall opioid prescribing among patients with chronic, non-cancer pain in a rural family medicine clinic: a controlled cross-over trial. J Am Board Fam Med.

[41] Zgierska AE, Robinson JM, Lennon RP (2020). Increasing system-wide implementation of opioid prescribing guidelines in primary care: findings from a non-randomized stepped-wedge quality improvement project. BMC Fam Pract.

[42] Samet JH, Tsui JI, Cheng DM (2021). Improving the delivery of chronic opioid therapy among people living with human immunodeficiency virus: a cluster randomized clinical trial. Clin Infect Dis.

[43] Keller S, Bann CM, Dodd SL, Schein J, Mendoza TR, Cleeland CS (2004). Validity of the brief pain inventory for use in documenting the outcomes of patients with noncancer pain. Clin J Pain.

[44] Zigmond AS, Snaith RP (1983). The hospital anxiety and depression scale. Acta Psychiatr Scand.

[45] Wittchen HU (1994). Reliability and validity studies of the WHO–Composite International Diagnostic Interview (CIDI): a critical review. J Psychiatr Res.

[46] Butler SF, Budman SH, Fernandez KC (2007). Development and validation of the current opioid misuse measure. Pain.

[47] Setnik B, Roland CL, Barsdorf AI, Brooks A, Coyne KS (2017). The content validation of the Self-Reported Misuse, Abuse and Diversion of Prescription Opioids (SR-MAD) instrument for use in patients with acute or chronic pain. Curr Med Res Opin.

[48] Robinson-Papp J, George MC, Wongmek A (2015). The quantitative analgesic questionnaire: a tool to capture patient-reported chronic pain medication use. J Pain Symptom Manage.

[49] Chesney MA, Ickovics JR, Chambers DB, et al. Self-reported adherence to antiretroviral medications among participants in HIV clinical trials: the AACTG adherence instruments. Patient Care Committee & Adherence Working Group of the Outcomes Committee of the Adult AIDS Clinical Trials Group (AACTG). AIDS Care. 2000;12(3):255–66. doi:10.1080/09540120050042891 10.1080/09540120050042891 10928201

[50] Hall MA, Zheng B, Dugan E (2002). Measuring patients' trust in their primary care providers. Med Care Res Rev.

[51] Dyer N, Sorra JS, Smith SA, Cleary PD, Hays RD. Psychometric properties of the Consumer Assessment of Healthcare Providers and Systems (CAHPS®) Clinician and Group Adult Visit Survey. Med Care. 50(suppl):S28–34. doi:10.1097/MLR.0b013e31826cbc0d 10.1097/MLR.0b013e31826cbc0d PMC3480671 23064274

[52] Berger BE, Ferrans CE, Lashley FR (2001). Measuring stigma in people with HIV: psychometric assessment of the HIV stigma scale. Res Nurs Health.

[53] Waugh OC, Byrne DG, Nicholas MK (2014). Internalized stigma in people living with chronic pain. J Pain.

[54] Brondolo E, Kelly KP, Coakley V (2005). The perceived ethnic discrimination questionnaire: development and preliminary validation of a community version1. J Appl Soc Psychol.

